# Reactive oxygen/nitrogen species contribute substantially to the antileukemia effect of APO866, a NAD lowering agent

**DOI:** 10.18632/oncotarget.27336

**Published:** 2019-11-19

**Authors:** Anne-Julie Cloux, Dominique Aubry, Mathieu Heulot, Christian Widmann, Oussama ElMokh, Francesco Piacente, Michele Cea, Alessio Nencioni, Axel Bellotti, Karima Bouzourène, Maxime Pellegrin, Lucia Mazzolai, Michel A. Duchosal, Aimable Nahimana

**Affiliations:** ^1^Central Laboratory of Hematology, University Hospital of Lausanne, Lausanne, Switzerland; ^2^Service of Hematology, University Hospital of Lausanne, Lausanne, Switzerland; ^3^Department of Physiology, University of Lausanne, Lausanne, Switzerland; ^4^Department of Internal Medicine, University of Genoa, Genoa, Italy; ^5^Division of Angiology, Heart and Vessel Department, Lausanne University Hospital, Lausanne, Switzerland

**Keywords:** tumor cell death, parthanatos, non-apoptotic death, APO866/FK866, NAD

## Abstract

APO866 is a small molecule drug that specifically inhibits nicotinamide phosphoribosyltransferase (NAMPT), a key enzyme involved in nicotinamide adenine dinucleotide (NAD) biosynthesis from the natural precursor nicotinamide. Although, the antitumor activity of APO866 on various types of cancer models has been reported, information regarding mechanisms by which APO866 exerts its cytotoxic effects is not well defined. Here we show that APO866 induces a strong, time-dependent increase in highly reactive ROS, nitric oxide, cytosolic/mitochondrial superoxide anions and hydrogen peroxide. We provide evidence that APO866-mediated ROS production is modulated by PARP1 and triggers cell death through mitochondria depolarization and ATP loss. Genetic or pharmacologic inhibition of PARP1 prevented hydrogen peroxide accumulation, caspase activation, mitochondria depolarization, ATP loss and abrogates APO866-induced cell death, suggesting that the integrity of PARP1 status is required for cell death. Conversely, PARP1 activating drugs enhanced the anti-leukemia activity of APO866

Collectively, our studies show that APO866 induces ROS/RNS productions, which mediate its anti-leukemia effect. These results support testing new combinatorial strategies to enhance the antitumor activities of APO866.

## INTRODUCTION

The renewed interest in the potential anticancer benefit of targeting tumor metabolism has led to an intense development of nicotinamide adenine dinucleotide (NAD) biosynthesis inhibitors [1–3]. NAD is an essential coenzyme in numerous intracellular redox reactions, including those that allow ATP (adenosine triphosphate) production (i.e. glycolysis and mitochondrial respiration). In addition, NAD is the substrate of several important NAD-degrading enzymes including poly (ADP-ribose) polymerases (PARPs) and sirtuins [4], that, in turn, are involved in crucial biological processes, such as genomic stability, apoptosis, aging, stress resistance, and metabolism [5–7]. In mammals, NAD can be synthesized through three main biochemical pathways from five distinct precursors: (i) tryptophan through the so-called *de novo* pathway; (ii) nicotinic acid (NA, or its related riboside form) through the Preiss-Handler pathway; or (iii) nicotinamide (NAM, or its related riboside form) through the salvage pathway. NAM/NA are the most important available NAD precursor in mammals [8–10]. NAMPT is the rate-limiting enzyme that catalyzes the phosphoribosylation of NAM to produce nicotinamide mononucleotide (NMN) [11, 12]. NMN is subsequently converted to NAD by NMN adenylyltransferases. Cancer cells have an increased need of NAD compared to normal cells, since most cancer cells exhibit a sustained PARP activation due to DNA damage and genomic instability [13, 14] and have higher energy demands [15]. Thus, tumor cells are more vulnerable to NAD depletion than normal cells [1, 16]. This observation has led to the development of NAMPT inhibitors, which indeed exhibit mechanism-based efficacy against a wide range of human solid tumors and blood cancers [2, 16–23]. Exposure of cancer cells to NAMPT inhibitors strongly decreases NAD cell content, followed by ATP decline that ultimately leads to cell death. NAMPT inhibitor-induced cell death occurs either in caspase-dependent or –independent manner, and is associated with reactive oxygen species (ROS) production, mitochondrial dysfunction, and autophagy [1, 16, 24–28]. However, the role of reactive oxygen/nitrogen species (ROS(29)/RNS) productions in NAMPT inhibitor-mediated cytotoxicity is not defined.

ROS are a group of small, short-lived and highly reactive oxygen molecules [30] containing one or more unpaired electrons [31, 32]. ROS include molecules such as oxygen radicals [superoxide anion (O_2_^-•^), hydroxyl radicals (OH^•^), peroxyl radicals (RO_2_^•^) and alkoxyl radicals (RO^•^)] and non-radicals such as hydrogen peroxide (H_2_O_2_) hypochlorous acid (HOCl), ozone (O_3_) and singlet oxygen (^1^O_2_) [30, 33, 34]. RNS are small molecules that include nitric oxide radical (NO^•^), peroxinitrite (ONOO^-^), nitrogen dioxide radical [14] (NO_2_^•^), other oxides of nitrogen and products arising when NO^•^ reacts with O_2_^-•^, RO^•^ and RO_2_^•^. Under physiologic conditions and at low/moderate concentrations, ROS/RNS play a central role in various cellular functions such as gene transcription, cell-cycle regulation and cell proliferation [29, 32, 35–37]. However, the overproduction of ROS/RNS results in the oxidation/nitrosylation of cell constituents such as proteins, DNA and lipids [30] that leads to cell dysfunction and ultimately to cell death [29, 30, 33].

In the present study, we evaluated ROS/RNS production in hematopoietic malignant cells treated with APO866, a very potent NAMPT inhibitor, and explored the role of ROS/RNS generation in APO866-induced cell death using pharmacological and genetic tools. We show that exposure of leukemia cells to APO866 leads to a tremendous increase in various types of ROS/RNS in a dose- and time-dependent manner. Excessive ROS/RNS release contributes to APO866-induced cell death through mitochondria depolarization and requires the integrity of PARP1 status. Finally, PARP1 activating chemotherapeutic drugs strongly potentiate the anti-leukemia activity of APO866.

## RESULTS

### APO866 dramatically increases ROS/RNS levels in hematological malignant cells

**Figure 1:**
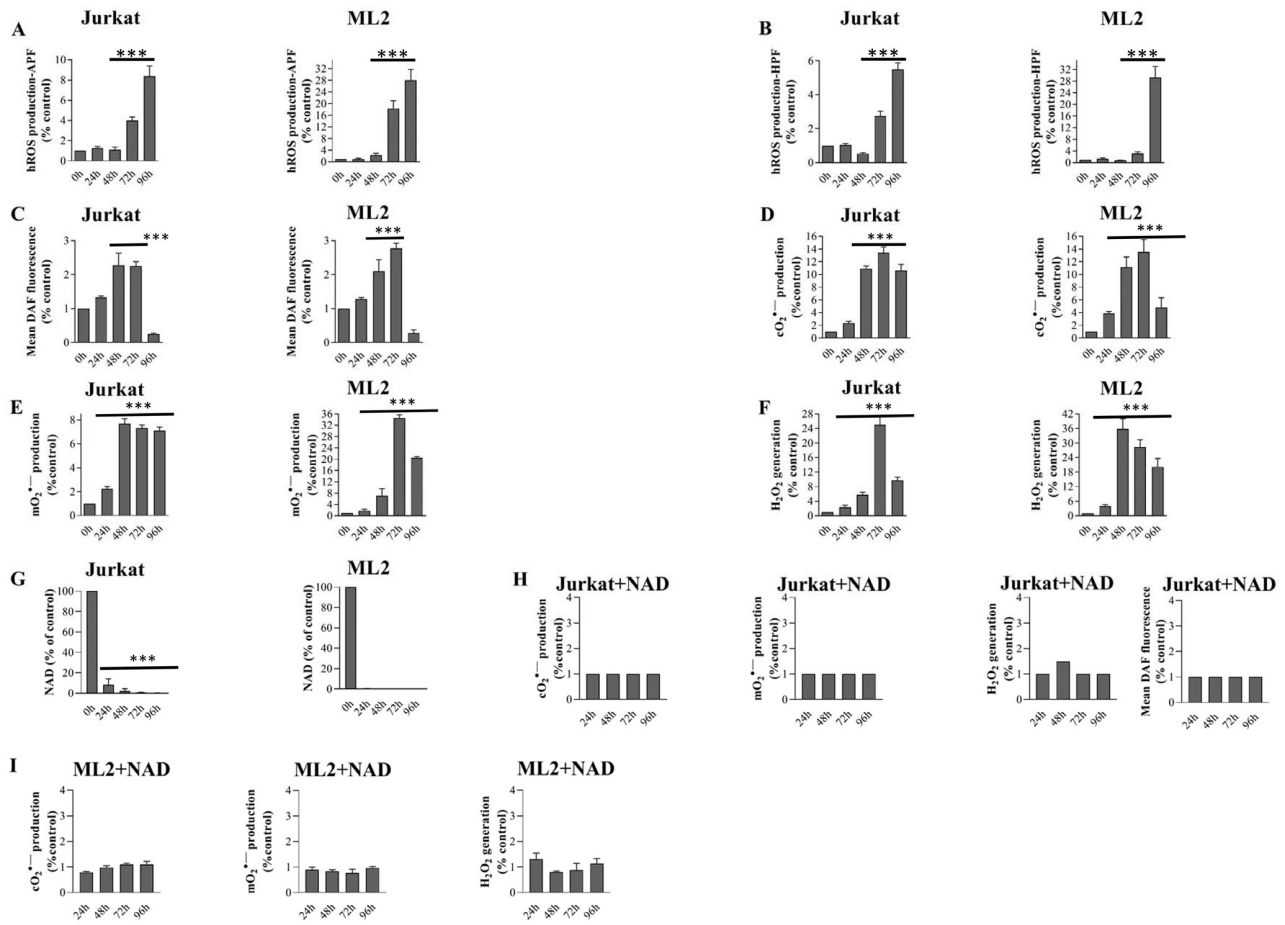
APO866 depletes NAD content that leads to dramatic increases ROS/RNS levels in hematological malignant cells. Time dependent detection of ROS/RNS productions in Jurkat and ML2 cells treated with APO866 (10 nM) with (**A**–**F**) or without (**H**–**I**) exogenous NAD. Highly reactive ROS (A–B), NO (C and H), Cytosolic (D, H and I), mitochondrial superoxide (E, H and I) and hydrogen peroxide (F, H and I) were detected by flow cytometry using APF, HPF, DHE, MitoSox, and DCFDA fluorescent probe, respectively. (**G**) Total intracellular NAD content was measured and normalized to relative protein content and expressed compared to untreated cells. Data are mean ± SD, *n* = 3; ^***^ *p* < 0.001 (vs. untreated cells).

In our previous studies, we reported the association between APO866 treatment and cO_2_, mO_2_, H_2_O_2_ accumulations [26, 38, 39]. Now, we provide a global view of the ability of APO866 to generate various type of ROS/RNS in hematopoietic malignant cells. We extended our investigation to the generation of highly reactive ROS (hROS) and NO in APO866-treated hematological malignant cells. hROS/NO are known to be detrimental to cells, since they chemically modify lipids, proteins, and nucleic acids. We took advantage of recently developed probes [Aminophenyl fluorescein (APF), hydroxyphenyl fluorescein (HPF), in addition to 5,6-Diaminofluorescein diacetate (DAF-2/DA)] that detect hROS and NO, respectively [40]. Two unrelated leukemia cell lines, Jurkat and ML-2 cells, were treated with or without APO866 for various time periods and hROS/NO levels were detected by flow cytometry and by specific sensitive probes. The exposure of hematopoietic malignant cells to APO866 was found to lead to a statistically significant increase in hROS and NO levels in a time-dependent manner until 96 hours (Figure 1A–1C). In parallel assays, we also confirmed our previous study, showing that, over time, APO866 induced strong increase in cO_2_, mO_2_ and H_2_O_2_ in both hematological malignant cell lines exposed to the drug (Figure 1D–1F). Next, we examined whether ROS/RNS production is due to NAD depletion. To this end, we first measured the intracellular NAD content in Jurkat and ML2 cells treated with or without APO866. As shown in Figure 1G, NAD depletion precedes ROS/RNS productions (Figure 1A–1F) in APO866-treated hematopoietic malignant. To confirm that oxidative/nitrosative stress in APO866-treated leukemia cells is a consequence of NAD depletion (induced by APO866), we evaluated ROS/RNS productions in APO866-treated leukemia cells in the presence of exogenous NAD. The supplementation with exogenous NAD allowed to fully inhibit ROS/RNS productions in APO866-treated leukemia cells (Figure 1H and 1I), which strongly suggests that NAD depletion is involved in ROS/RNS productions.

Collectively, these data strongly suggest that APO866 depletes intracellular NAD content, thereby leading to the generation of various types of ROS/RNS.

### ROS/RNS generations are required for APO866-mediated cell death

**Figure 2:**
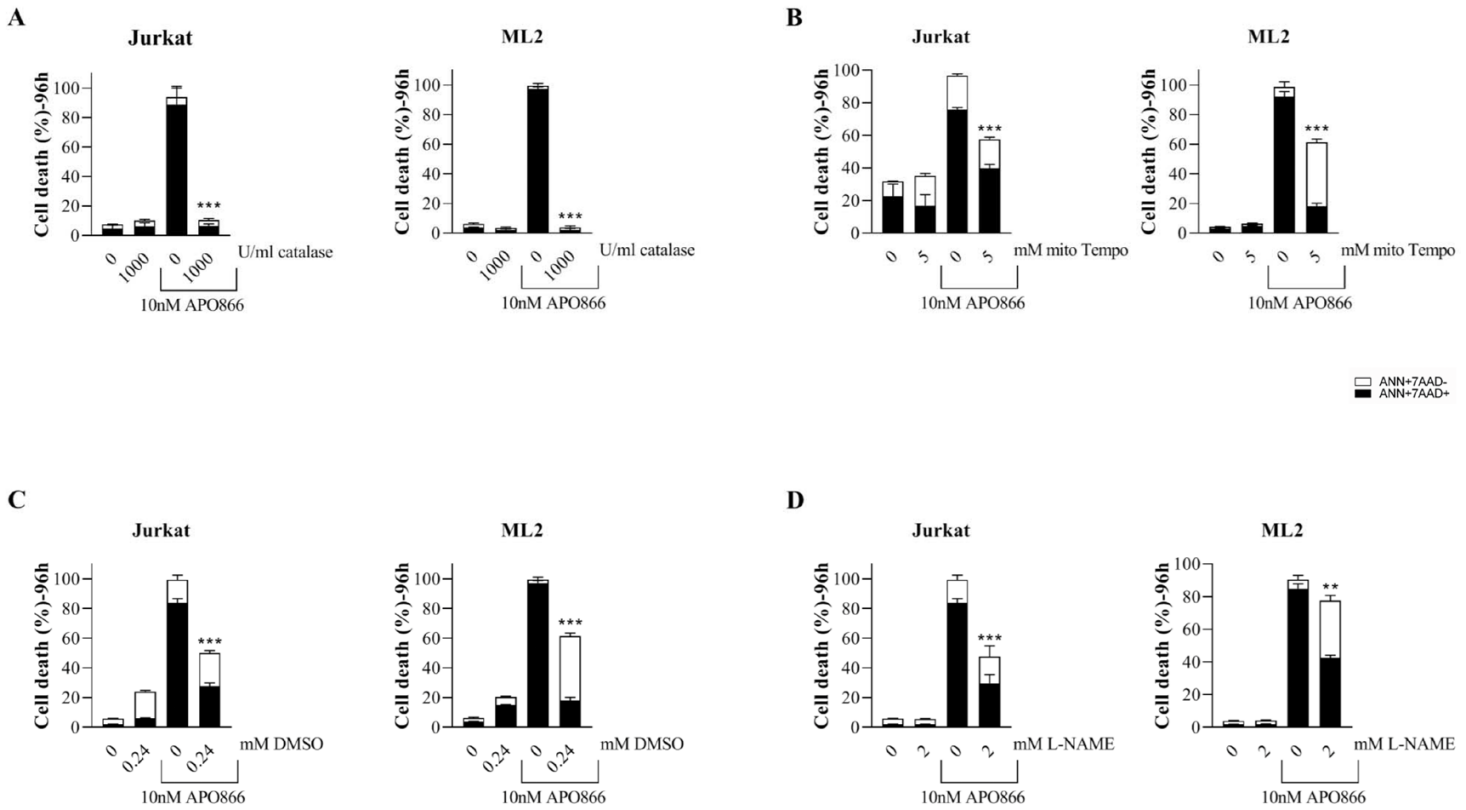
Scavenging ROS/RNS attenuates APO866-mediated cell death in leukemia cells. Jurkat and ML2 cells were incubated with or without (**A**) catalase (H_2_O_2_ scavenger, 1000 U/ml), (**B**) mitoTempo (mitochondria superoxide scavenger, 5 mM), (**C**) DMSO (hydroxyl radical scavenger, 0.24 mM), or (**D**) L-NAMAE (NO scavenger, 2 mM) with or without APO866 (10 nM). Cell death after 96 hours of drug exposure was assessed by flow cytometry using ANXA5 and 7AAD stainings. The percentage of early apoptotic cells (ANN+ 7AAD−) are shown as white columns and that of late apoptotic cells (ANN+ 7AAD+) are shown as solid black columns. Data are mean ± SD, *n* ≥ 3; ^**^ *P* < 0.01; ^***^ *P* < 0.001.

**Figure 3:**
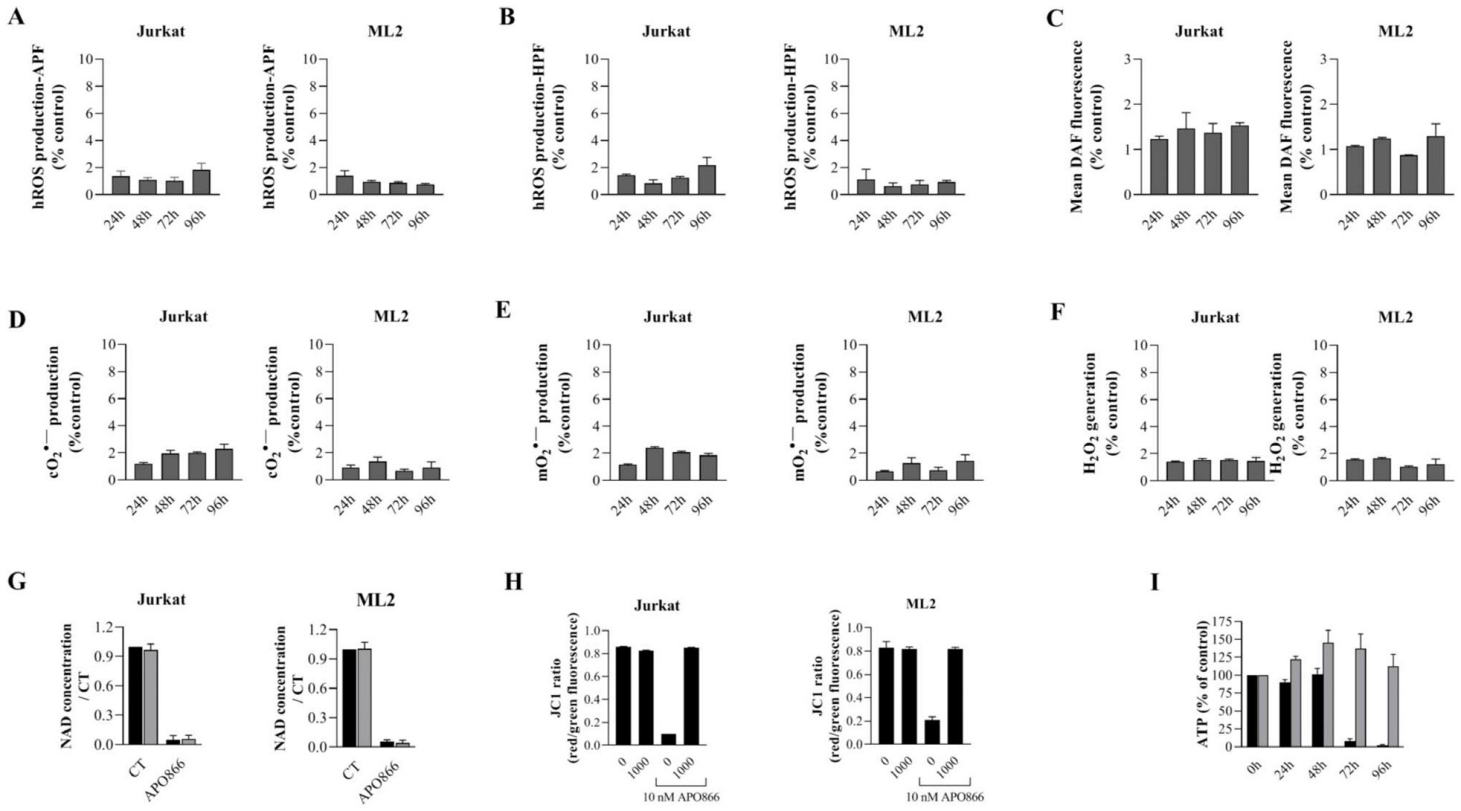
Catalase supplementation abrogates ROS/RNS production, loss of mitochondria membrane potential and ATP, but not NAD depletion in APO866-treated hematological malignant cells. Time dependent detection of ROS/RNS productions in Jurkat and ML2 cells treated with APO866 (10 nM) in presence of exogenous addition of catalase. Highly reactive ROS (**A**–**B**), NO (**C**), Cytosolic (**D**), mitochondrial superoxide (**E**) and hydrogen peroxide (**F**) were detected as described in Figure 1. Detection of NAD and ATP cell content (**G** and **I**), as well as mitochondrial depolarization (**H**) in APO866-treated Jurkat and ML2 cells in presence (or absence) of catalase (1000 U/ml) were assessed as described in Method section. Total intracellular NAD and ATP contents were measured and normalized to relative protein content and are expressed as a ratio compared to that of untreated Jurkat/ML2 cells.

Next, we evaluated the contribution of ROS/RNS productions in APO866-induced cell death in leukemia cells. Hematopoietic malignant cells were exposed to APO866 in presence or absence of different ROS scavengers and cell death was monitored. Catalase, Mito-TEMPO, dimethyl sulfoxide (DMSO) and N-Nitroarginine methyl ester (L-NAME) were used as a scavengers for H_2_O_2_, mO_2_, OH radical and NO; respectively. Scavenging ROS/RNS generations (Figure 2A–2D) significantly attenuates APO866-induced cell death, with catalase providing a complete protection from APO866-induced cell death. To confirm that cell death protection by ROS/RNS scavengers indeed reflects the suppression of oxidative/nitrosative stress, we examined ROS production in APO866-treated leukemia cells in presence/or absence of catalase. As expected, pretreatment with catalase completely abrogated APO866-induced ROS production in all tested malignant cells (Figure 3A–3F). Notably, NAD depletion was still found to occur in APO866-treated leukemia cells despite the presence of exogenous catalase (Figure 3G), indicating that the contribution of ROS/RNS productions to APO866-induced cell death occurs downstream of NAD depletion. We previously reported the association between APO866-induced cell death and ROS productions and hypothesized that the high level of APO866-induced ROS levels were responsible for mitochondria damage, as highlighted by (i) the loss in mitochondria transmembrane potential, (ii) ATP depletion and finally (iii) cell death [16, 26]. Here, we examined whether scavenging ROS/RNS accumulation in APO866-treated leukemia cells would prevent mitochondria depolarization. Hematopoietic malignant cells were pre-treated with (or without) catalase before incubation with (or without) APO866 and mitochondrial transmembrane potential (ΔΨ_m_) was assessed using JC-1 staining and flow cytometry. In line with previous studies [16, 26], APO866 was found to dissipate ΔΨ_m_ in leukemia cells. In contrast, prevention of ROS/RNS accumulations by catalase supplementation abrogated mitochondria depolarization, suggesting that oxidative stress plays a key role in APO866-mediated mitochondria depolarization (Figure 3H). One of the consequences of mitochondria depolarization is ATP loss. Then, we assessed ATP cell content in APO866-treated leukemia cells in presence or absence of catalase. In agreement with our observation, exogenous addition of catalase fully prevents the ATP depletion induced by APO866 treatment (Figure 3I). These results also indicate that the protective effects of ROS/RNS scavengers in APO866-treated leukemia cells rely on the abrogation of mitochondrial depolarization and thereby preventing the ATP loss.

Taken together, these results clearly indicate that ROS/RNS production is a consequence of NAD depletion and that it is involved in the ΔΨ_m_ dissipation that, in turn, leads to ATP loss contributing to the anti-leukemia activity of APO866.

### APO866-mediated leukemia cell death requires PARP1 integrity

**Figure 4:**
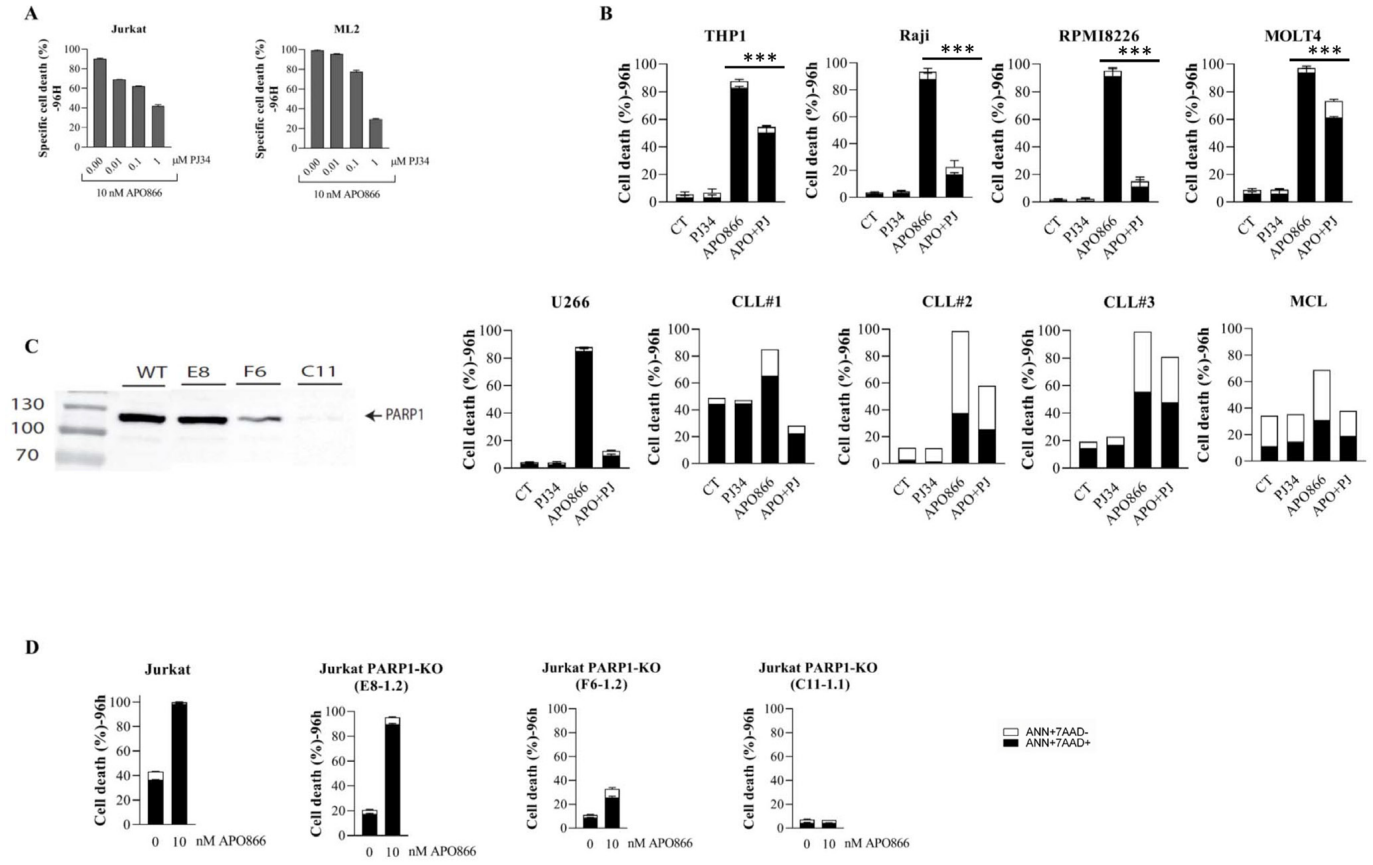
The integrity of PARP1 status is required for the anti-leukemia activity of APO866. Cell lines and primary cells from different hematological malignancies were treated without or with 10 nM of APO866 in the presence or absence of a pharmacological inhibition (**A**–**B**) or genetic deletion (**D**) of PARP1 and cell death assessed as described in Figure 2. PARP1 was knocked out (KO) in wild-type (WT) Jurkat cells using CRISPR/Cas9 technology. Loss of expression was confirmed by Western blotting (**C**).

The deleterious effects of hROS/NO are also mediated via DNA damage, resulting in PARP1/caspase activations and ultimately leading to cell death [34, 35]. To assess whether PARP1 is involved in APO866-induced cell death, cells from different hematologic malignancies were pre-incubated with a potent PARP1 inhibitor (PJ34) prior APO866 treatment. PJ34 significantly decreases APO866-mediated cell death in dose-dependent manner (Figure 4A), suggesting the implication of PARP1 activation in the anti-leukemia effects of the NAMPT inhibitor. To assess the generality of our observation and demonstrating that it is not restricted to one or two cell lines, we extended our investigation to additional leukemia, lymphoma and multiple myeloma cell lines and to primary B-CLL and MCL cells from patients. PARP1 inhibition confers a significant protective effect against APO866-induced cytotoxicity in all of the blood cancer cells that were tested (Figure 4B).

**Figure 5:**
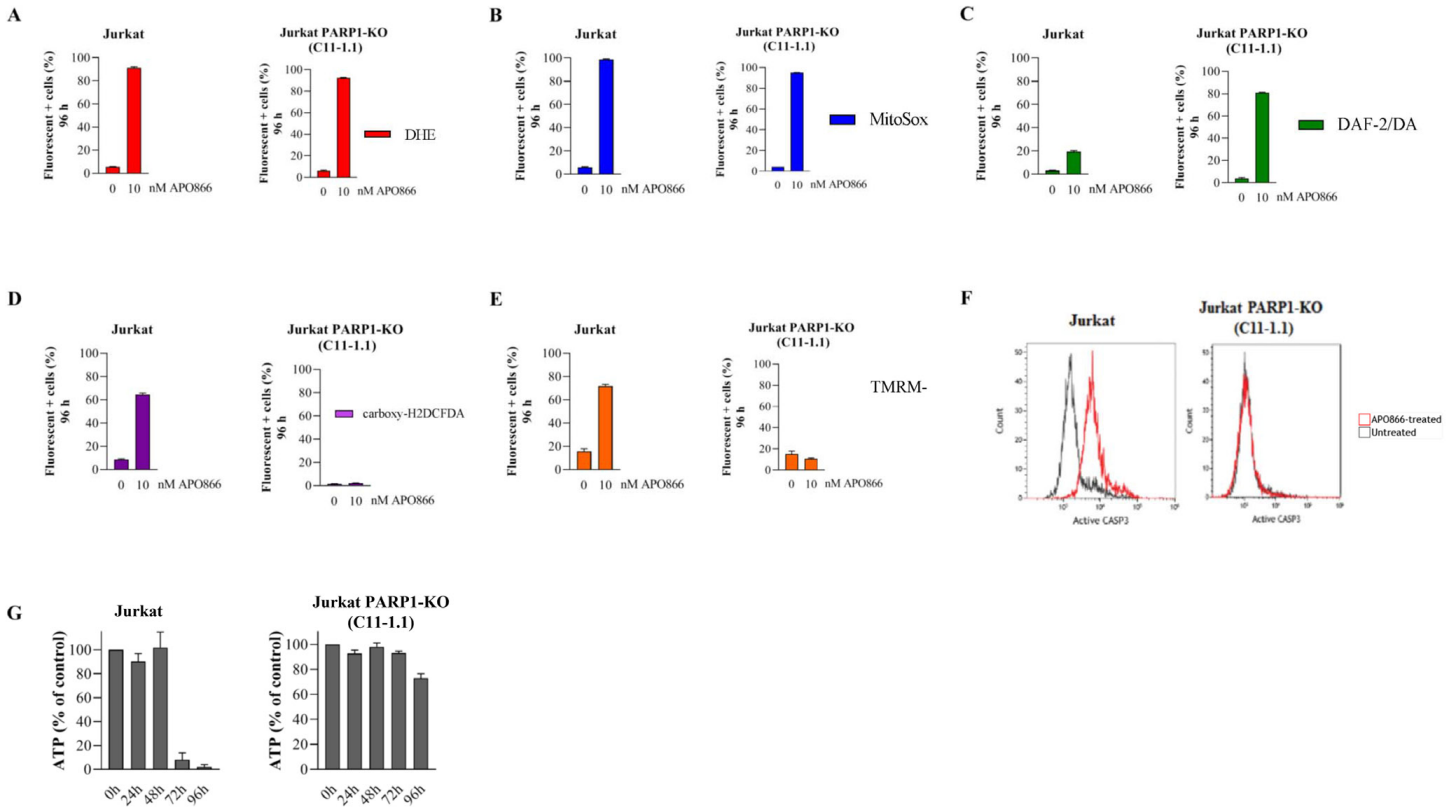
Deletion of PARP1 fully inhibits H_2_O_2_ production, mitochondrial depolarization, caspase activation, and loss of ATP cell content in APO866-treated Jurkat cells. Detection of cytosolic (**A**), mitochondrial (**B**) superoxide anions, NO (**C**), H_2_O_2_ generation (**D**) and MMP (**E**), and ATP cell content (**G**) in APO866-treated WT or KO Jurkat cells were monitored as described in Figure 1. (**F**) Caspase 3 activation was assessed in WT (or PARP1KO) Jurkat cells treated with 10 nM APO866 for 96 h using a fluorescent specific probe for activated forms of CAS P3 and flow cytometry.

In principle, pharmacological inhibition may have off target effects. To complement the pharmacological PARP1 inhibition and to verify its contribution in the anti-leukemia effects of APO866, we generated PARP1 knockout Jurkat cells (Figure 4C). Sensitivity of Jurkat cells to APO866 correlated with PARP1 levels (Figure 4D). Notably, in the PARP1-KO clone C11-1.1, in which PARP1 expression was virtually abrogated, we observed a complete resistance to APO866. Thus, the latter clone was used for further experiments. We then examined whether ROS/RNS accumulation in APO866-treated hematopoietic tumor cells depends on PARP1 status. To this end, we evaluated the effect of PARP1 deletion on ROS production in APO866-treated malignant cells. Wild-type (WT) or PARP1-KO Jurkat cells were exposed to APO866 for various time and intracellular ROS production was measured using DHE (for cO_2_), MitoSOX (mO_2_), carboxy-H2DCFDA (H_2_O_2_), and DAF2/DA (NO) and flow cytometry. Deletion of PARP1 had no effect on cO_2_, mO_2_ and NO production (Figure 5A–5C), but fully prevented the accumulation of H_2_O_2_ (Figure 5D). Since H_2_O_2_ is deleterious to mitochondria, we next examined the effect of PARP1 KO on mitochondria depolarization. Deletion of PARP1 prevented ΔΨ_m_ loss in response to APO866 (Figure 5E). Mitochondria depolarization is expected to release cytochrome c, which in turn leads to caspase activation and, consequently, to DNA degradation and ATP depletion. PARP1 deletion fully prevented caspase activation and ATP loss in APO866-treated leukemia cells (Figure 5F–5G). To get further insights into the contribution of PARP1 in the metabolism of oxidative stress, we carried out a pilot study by comparing the expression levels of 84 genes that regulate oxidative stress (including antioxidant genes) in WT versus PARP1-KO Jurkat cells in presence or absence of APO866. In agreement with the above data, genetic deletion of PARP1 showed that of the 84 genes analyzed, changes in mRNA (fold change > 1.5) were detected in 21 genes: 5 upregulated and 16 downregulated (Table 1). Most of downregulated genes are involved in the production of oxidative stress. In addition, treatment of PARP1-KO Jurkat cells with APO866 resulted in upregulation of 6 powerful antioxidant genes, including CAT and genes known to increase tumor cell survival such SIRT2 and UCP2 (Table 2). Taken together, these results suggest that oxidative stress (including H_2_O_2_ production) is modulated by PARP1, and that H_2_O_2_ plays a key role in the mitochondrial depolarization, caspase activation, ATP loss, and cell death.

**Table 1:** Fold changes for 21 PCR-significant genes

Gene symbol	GenBank accession no	Description	Fold change (*P* < 0.05)			
			WT vs PAPR1KO	WT vs PAPR1KO +APO	PARP1KO vs PARP1KO+APO	WT vs WT+APO
PXDN	NM_012293	Peroxidasin	2.03	4.06	1.67	–1.16
PNKP	NM_007254	Polynucleotide kinase 3’-phosphatase	1.87	3.66	1.64	–1.27
STK25	NM_006374	Serine/threonine kinase 25	1.85	3.61	1.67	1.39
SRXN1	NM_080725	Sulfiredoxin 1	1.59	4.35	2.29	1.31
ALOX12	NM_000697	Arachidonate 12-lipoxygenase	1.57	3.63	1.94	–1.43
MSRA	NM_012331	Methionine sulfoxide reductase A	–3.86	–2.83	1.14	–1.11
MBL2	NM_000242	Mannose-binding lectin 2	–2.77	–2.11	1.1	–1.21
MB	NM_005368	Myoglobin	–2.77	–2.11	1.1	–1.21
GPX6	NM_182701	Glutathione peroxidase 6	–2.77	–2.11	1.1	–1.21
ALB	NM_000477	Albumin	–2.77	–2.11	1.1	–1.21
GPX5	NM_001509	Glutathione peroxidase 5	–2.77	–2.11	1.1	–1.21
SFTPD	NM_003019	Surfactant protein D	–2.77	–2.11	1.1	–1.21
PTGS2	NM_000963	Prostaglandin-endoperoxide synthase 2	–2.77	–2.11	1.1	–1.21
NOX5	NM_024505	NADPH oxidase, EF-hand calcium binding domain 5	–2.77	–2.11	1.1	–1.21
NOX4	NM_016931	NADPH oxidase 4	–2.77	–2.11	1.1	–1.21
APOE	NM_000041	Apolipoprotein E	–2.1	–1.49	1.18	–1.3
LPO	NM_006151	Lactoperoxidase	–2.01	–2.11	–1.25	–1.21
MT3	NM_005954	Metallothionein 3	–1.67	–1.99	–1.42	–1.09
GPX3	NM_002084	Glutathione peroxidase 3	–1.62	1.09	1.49	–1.38
PRDX3	NM_006793	Peroxiredoxin 3	–1.58	1.01	1.34	1.21
AOX1	NM_001159	Aldehyde oxidase 1	–1.53	–1.04	1.22	–1.06

**Table 2:** Fold changes for 9 antioxidant significantly upregulated genes in APO866-treated PARP1KO leukemic cells

Gene symbol	GenBank accession no	Description	Fold change (*P* < 0.05)			
			WT vs PAPR1KO	WT vs PAPR1KO +APO	PARP1KO vs PARP1KO+APO	WT vs WT+APO
SIRT2	NM_012237	Sirtuin 2	–1.07	3.76	3.37	1.44
GSR	NM_000637	Glutathione reductase	1.13	4.29	3.19	1.01
UCP2	NM_003355	Uncoupling protein 2	–1.82	1.62	2.48	–1.68
DHCR24	NM_014762	24-dehydrocholesterol reductase	1.22	3.27	2.24	1.32
TXNRD1	NM_003330	Thioredoxin reductase 1	–1.06	2.2	1.99	–1.1
SOD2	NM_000636	Superoxide dismutase 2, mitochondrial	1.06	2.2	1.73	1.3
CAT	NM_001752	Catalase	–1.19	1.7	1.7	1.35
VIMP	NM_203472	Selenoprotein S	–1.25	1.52	1.58	1.21
PREX1	NM_020820	Phosphatidylinositol-3,4,5-trisphosphate-dependent Rac exchange factor 1	1.06	1.91	1.5	1.01

### PARP1 activating agents sensitize leukemia cells to APO866 treatment

**Figure 6:**
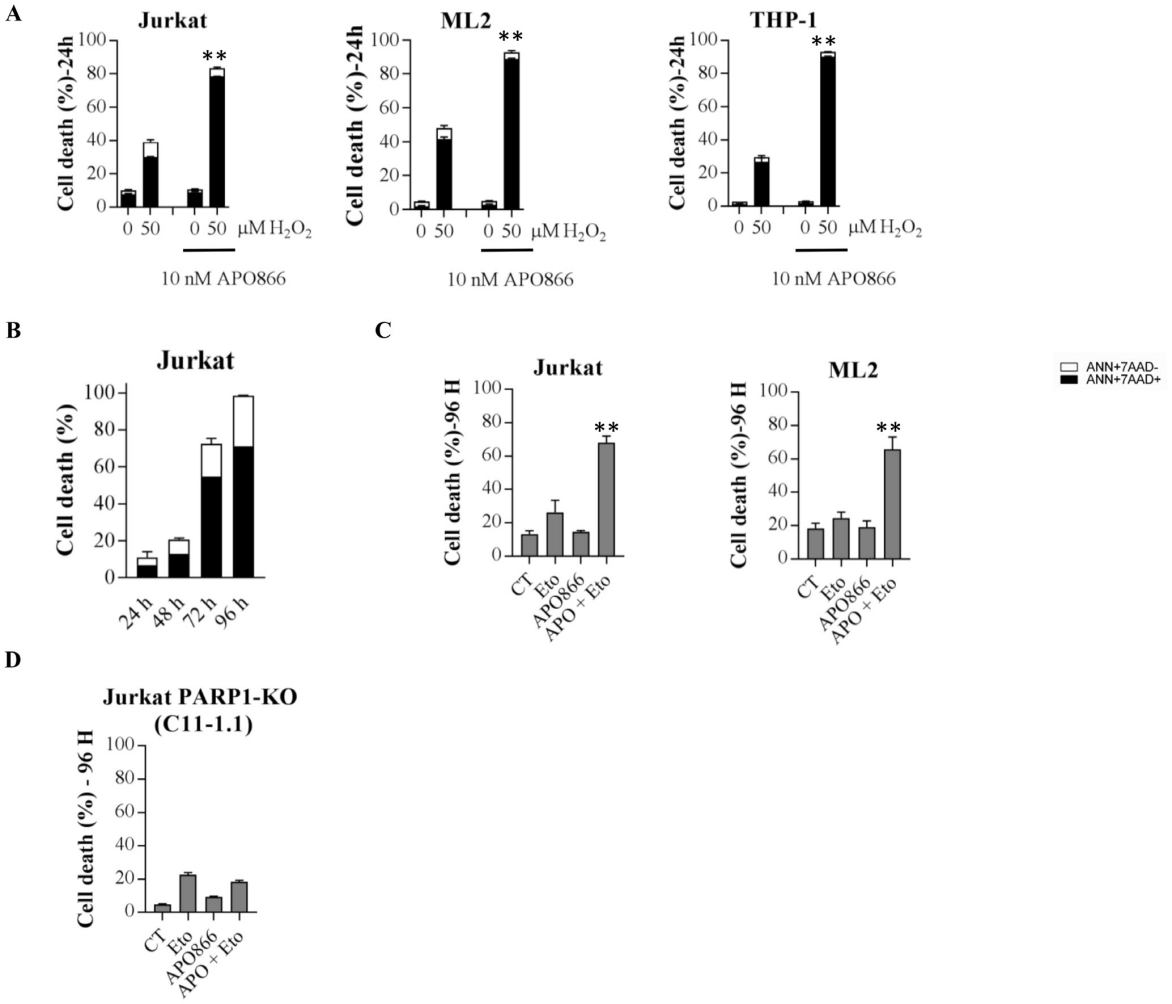
Exogenous supplementation of H_2_O_2_ or etoposide potentiates the anti-leukemia activity of APO866. APO866-treated WT or PARP1 KO cells from different human leukemia exposed to H_2_O_2_ (**A**) or not (**B**), or etoposide (**C** and **D**) alone or in combination for 24 hours (H_2_O_2_) or 96 hours (Etoposide). Cell death was assessed as described in Figure 2. Data are mean ± SD, *n* = 3; ^**^ *p* < 0.001 (drug combination vs. each drug alone).

Understanding the molecular mechanisms involved in APO866-induced cell death enabled us to establish a rationale for combinatorial strategy with APO866 that could increase its cytotoxic activity in hematopoietic cancer cells. Specifically, based on the above-mentioned data, we hypothesized that chemotherapeutic agents that activate PARP1 (such as alkylating agents) or that boost ROS production could sensitize leukemia cells to APO866. To test our hypothesis, we treated hematopoietic malignant cells with (or without) APO866 in presence (or absence) of exogenously added H_2_O_2_ and assessed cell death as described above. Exogenous supplementation of H_2_O_2_ in APO866-treated leukemia cells, significantly increased the anti-leukemia activity of APO866 (Figure 6A) and shortened the time required to achieve cytotoxic activity from 96 hours to 24 hours (Figure 6B), indicating that ROS-producing chemotherapeutic drugs could sensitize leukemia cells to APO866. Next, we evaluated the ability of etoposide (a PARP1 activating drug through topoisomerase II inhibition) to sensitize leukemia cells to APO866. Hematopoietic malignant cells were exposed to etoposide alone or to the combination of etoposide and APO866 for 96 hours and subsequently measured cell death as described above. Etoposide plus APO866 treatment significantly increased cell death as compared to each drug alone in all cells tested (Figure 6C). We further evaluated the effects of the combined action of etoposide (or H_2_O_2_) and of APO866, by calculating a CI for each combination. The CI for etoposide/H_2_O_2_ and APO866 was < 1 for all of the tested leukemia cell lines, indicating a synergistic effects of this combination in these malignant cells (Tables 3 and 4). Collectively, these data indicate that PARP1 activating drugs could potentiate the anti-leukemia activity of APO866. Finally, to assess whether sensitization of leukemia cells to APO866 by etoposide involves PARP1 activity, PARP1-KO malignant cells were exposed to etoposide alone or etoposide plus APO866 for 96 hours before assessing their viability. Deletion of PARP1status abrogated the synergistic effect of combined APO866 and etoposide (Figure 6D).

**Table 3:** Synergistic effect of H2O2 and APO866 on different leukemic cells

Cell line	Jurkat	ML2	THP1
**CI**	0.4	0.5	0.3

**Table 4:** Synergistic effect of Etoposide and APO866 on different leukemic cells

Cell line	Jurkat	ML2
**CI**	0.3	0.1

Abbreviations: CI, cooperative index.

## DISCUSSION

Here we show that the NAMPT inhibitor APO866 is a very potent inducer of different types of ROS/RNS, including cO_2_/mO_2_, H_2_O_2_, NO and hROS and that these strongly contribute to its anti-leukemia activity. ROS/RNS accumulation under the modulation of PARP1 triggers mitochondrial depolarization and ATP depletion, and finally APO866-induced cell death. In line with this model, supplementation with exogenous ROS scavenging agents, such catalase or inhibition of PARP1, were found to protect from APO866-induced cytotoxicity. To our knowledge, this is the first study to report such diversity of ROS/RNS productions in APO866-treated leukemia cells. Our data indicate that in response to APO866 treatment, mO_2_, cO_2_ and NO production is independent of PARP1 status, whereas H_2_O_2_ generation depends on the PARP1 integrity status. Of interest, PARP1 proficiency in cancer cells is required for APO866 to be cytotoxic in these cells. The present study also highlights the role of PARP1 in oxidative stress production, the cumulative anti-leukemia potentials of APO866 and chemotherapeutic drugs that active PARP1, such as etoposide.

ROS accumulation in APO866-treated hematological malignant cells is in agreement with previous studies reporting ROS production in malignant cells treated with NAMPT inhibitors [41, 42]. The enhanced ROS production in APO866-treated malignant cells could be explained, at least in part, by the fact that, by depleting intracellular NAD content, APO866 affects the NAD(P)/NAD(P)H ratio as reported in our previous study [43], thus altering the cellular redox status. The impaired ability of leukemia cells treated with APO866 to perform ROS scavenging would then result in enhanced oxidative/nitrosative stress highlighted by the strongly increased ROS/RNS levels. In this regard, the NADP(H) cell content has been shown to contribute to the variability in metabolic response to NAD depletion [42]. We clearly demonstrated the involvement of ROS production in APO866-mediated cytotoxicity. Specifically, our data strongly suggest that APO866 induces NAD depletion that leads to oxidative stress mode shown by the production of various types of ROS/RNS, particularly H_2_O_2_, which triggers cell death via the mitochondrial apoptotic pathway. Our result suggest also that PARP1 status plays an important role in oxidative stress and cell death. To explain this finding, we provide strong evidence that PARP1 regulates the expression level of oxidative stress genes. In line with this observation, we showed that genetic inhibition of PARP1 resulted in downregulation of several genes involved in oxidative stress metabolism. Treatment of PARP1-KO leukemic cells with APO866 led also to an upregulation of antioxidant genes including (catalase and other genes DHCR24, GSR, UCP2, SIRT2…) that are known to play a crucial role in drug resistance in different types of cancer cell, which could provide a plausible explanation on how PARP1-KO leukemic cells displayed less sensitivities to APO866.

We cannot rule out that the accumulation of ROS (Figure 1A–1F) due to NAD depletion (Figure 1G) could lead to DNA damage [44] thus activating PARP1 [43]. The activation of PARP1 could contribute to the exacerbation of NADP(H) cell content depletion [43], which in turn will lead to the reduction in the antioxidant cell capacity through depletion of potent antioxidant enzymes (Tables 1 and 2) such, superoxide dismutase, and heme oxygenase 1 [45] and ultimately result in the observed increase in H_2_O_2_ production (Figure 1F). To demonstrate that NAD depletion is the primary cause of ROS accumulation, exogenous addition of NAD full inhibited ROS/RNS production (Figure 1H and 1I). Alternativelly and not mutually exclusive, we showed that ROS/RNS production provokes the disruption of mitochondria ΔΨ_m_ (Figure 3H). Mitochondria play a crucial role in energy metabolism and, perhaps even more importantly, in regulation of cell death [37]. Excessive ROS production can lead to mitochondria dysfunction, culminating in ATP depletion (Figure 3I) and, ultimately, in cell demise through the release of intra-mitochondrial enzymes involved in cell death such apoptotic protease activating factor 1 (apaf-1), cytochrome c, apoptosis-inducing factor (AIF), endoG. In line with the hypothesis, we showed that APO866 induces high level of ROS/RNS productions, and that H_2_O_2_ acts downstream of PARP1 in mediating APO866’s antilekeumia effects, suggesting that H_2_O_2_ accumulation is modulated by PARP1 status. The contribution of PARP1 in the depletion of antioxidant leukemia cell capacity (catalase; heme oxygenase-1) is suggested by the fact that genetic deletion of PARP1 indeed fully blunted H_2_O_2_ generation in APO866-treated cancer cells, via regulation of several genes involved in oxidative stress, including many antioxidant genes. In addition, we previously reported that APO866 is effective at depletion catalase [26, 43]. Blocking either ROS accumulation by exogenous addition of catalase or knocking out PARP1, were able to abrogate mitochondria depolarization, ATP loss and to fully inhibit cell death in response to APO866 treatment. Our results suggest that APO866 triggers parthanatos in leukemia cells. Parthanatos is a highly programed form of cell death, which occurs through the overactivation of PARP-1 [46]. Under normal conditions, PARP1 is involved in DNA repair. To maintain genomic homeostasis, PARP1 detects single strand DNA breaks, it uses NAD to catalyze the polyADP-ribosylation of PARP1 itself as well as of numerous other target proteins [5, 47]. This leads to the recruitment to the DNA damage sites of proteins that are critical for DNA repair [47]. However, PARP1 hyperactivation leads to NAD and ATP depletion and to the translocation of AIF from the mitochondria to the nucleus [48, 49]. In response to APO866, NAD depletion occurs upstream of PARP1 involvement. Based on our results, we cannot rule out the possibility that NAMPT inhibition may trigger other types of cell death in other cancer cells. In agreement with this observation, it was reported that NAD depletion could induce oncosis in non haematological cancer cells [50]. Further experiments are warranted to elucidate the key molecular events that control the cell death mechanisms triggered by NAMPT inhibitor-mediated NAD depletion and how does PARP1 to regulate oxidative stress genes. It is noteworthy to mention that although, we showed that PARP1 inhibition confers a protection from APO866-induced cell toxicity in a broad range of leukemia cells, it was reported elsewhere that NAMPT inhibition significantly enhanced the sensitivity of triple-negative (ER-, PR-, HER2-negative) breast cancer cells to olaparib (PARP1 inhibitor) treatment [51]. The increased sensitivity of TN breast cancer cells to olaparid in presence of NAMPT inhibitor could be due most probably to the defect in homologous recombination genes (such as loss-of-function BRCA1 or BRCA2 mutations).

We show that the chemotherapeutic drug, etoposide, that activates PARP1, or exogenous supplementation with H_2_O_2_ could be a successful strategy to sensitize leukemia cells to APO866. Particularly, such an approach is anticipated to be a valuable strategy as long as APO866-induced cell death is dependent on PARP1 activation (parthanatos). This observation is line with previous studies showing that the killing effects of NAMPT inhibitors could be increased by co-treatment with: (i) DNA-damaging drugs [25, 52] such etoposide, as cisplatin, 1-methyl-3-nitro-1-nitrosoguanidinium [53], fluorouracil [54] and (ii) ionizing radiations.

In conclusion, we demonstrated that APO866 induces the production of high levels of various types of ROS/RNS that depend on PARP1 integrity and playing a crucial role in its anti-leukemia activity. ROS/RNS accumulation triggers APO866-induced cell death through mitochondria depolarization, caspase activation and finally, to ATP depletion. We show that etoposide sensitizes leukemia cells to APO866 via PARP1 status. These findings suggest potential approaches to enhance the antitumor activity of APO866, by modulating the parthanatos pathway. Defining the molecular mechanisms underlying APO866-induced cytotoxicity is going to improve our understanding of the effects of NAD depletion in leukemia cells and it will aid in the development of novel anticancer therapy strategies.

## MATERIALS AND METHODS

### Cell lines and culture conditions

Seven hematological cancer cell lines were purchased from DSMZ (German Collection of Microorganisms and Cell Cultures) or ATCC and include Jurkat and Molt-4 (T-acute lymphoblastic leukemia); ML-2 and THP-1 (acute myeloid leukemia); RPMI8226 and U266 (multiple myeloma) and Raji (Burkitt lymphoma).

Primary cells from four patients were also analyzed. The ethics committee at the University of Lausanne approved study protocols. Primary cells were collected from peripheral blood (purity > 90%) from patients with mantle cell lymphoma (MCL; *n* = 1); and B-chronic lymphocytic leukemia (CLL; *n* = 3).

All cells were cultured in RPMI (Invitrogen AG, 61870-01) supplemented with 10% heat inactivated fetal calf serum (Amimed, 2-01F30-I) and 1% penicillin/streptomycin at 37°C (Amimed, 4-01F00-H) in a humidified atmosphere of 95% air and 5% CO_2_.

### Flow cytometer analyses

Various cellular effects induced by clinical grade APO866, (kindly provided by TopoTarget, Switzerland), on hematopoietic malignant cells were evaluated using a Beckman Coulter Cytomics Gallios flow cytometer and included following functional cell parameters: cell death, ROS/RNS productions, and mitochondrial membrane potential.

### Cell death analysis

APO866-induced cell death was determined using ANNEXIN-V (ANN; eBioscience, BMS306FI/300) and 7-aminoactinomycin D (7AAD; Immunotech, A07704) stainings as described by the manufacturer and analyzed using a flow cytometry. Dead cells were identified as ANN+ and/or 7AAD +. Specific cell death induced by drug was calculated using the following formula: percent cell death induced by drug = [(S - C) / (100 - C)] x 100; where S = treated sample cell death and C = untreated sample cell death.

### Assessment of mitochondrial membrane potential

MMP was determined using flow cytometry after cell staining with 5,5′,6,6′-tetrachloro-1,1′,3,3′-tetraethylbenzimididazolylcarbocyanine iodide (JC-1, Calbiochem, 420200-5). JC-1 is a cell permeant, fluorescent dye that readily accumulates in active mitochondria due to their relative negative charge. JC-1 accumulates in the mitochondria, showing green fluorescence at a low MMP and forming red fluorescent J-aggregates at higher MMP. A drop in MMP is indicated by a decrease in the ratio of the red signal to the green signal. Briefly, cells were cultured in the presence or absence of APO866 for 24 to 96 h. Cells were centrifuged, resuspended in phosphate-buffered saline (PBS) containing 5 μM JC-1, and were then incubated at 37°C for 15 min in the dark. The cells were washed twice with prewarmed PBS, and immediately analyzed using flow cytometry.

### Detection of cellular and mitochondrial reactive oxygen/nitrogen species (ROS/RNS)

Various type of ROS/RNS were determined in APO866- and control- treated hematopoietic malignant cells by flow cytometry using live-cell permeant specific fluorogenic probes. DHE as probe for detection of cytosolic superoxide anion (cO_2_^•-^), MitoSox as probe for detection of mitochondrial superoxide anion (mO_2_^•-^), carboxy-H_2_DCFDA as probe for detection of H_2_O_2_. APF and HPF as probes for detection of highly reactive oxygen/nitrogen species (hROS) that include singlet, hydroxyl radical, peroxynitrite anion, or hypochlorite anion. DAF-2/DA was used as probe for detection of intracellular NO. DHE is oxidized to red fluorescent ethidium by cytosolic superoxide and MitoSox is selectively targeted to mitochondria, where it is oxidized by superoxide and exhibits red fluorescence. 6-carboxy-2′,7′-dichlorodihydrofluorescein diacetate (carboxy-H_2_DCFDA) is cleaved by esterase to yield DCFH, a polar non-fluorescent product, but in presence of hydrogen peroxide, the latter is oxidized to green fluorescent product, dichlorofluorescent (DCF). APF and HPF reagents are nonfluorescent and produce bright green fluorescence upon reaction with hydroxyl radical or peroxynitrite anion, APF also reacts with the hypochlorite anion. DAF-2/Da is a membrane permeable, fluorescent that is hydrolyzed to DAF-2 by intracellular esterases and can be used as real-time indicator for NO level. For cell staining, cells were centrifuged and the pellets were resuspended in PBS with a final concentration of 5 μM for each probe. The mixture was incubated in the dark at 37°C for 15 min. Then, the cell suspension was analyzed using a flow cytometry within 20 min.

### NAD and ATP quantification

Cells (0.5 × 10^6^) in log growth phase were seeded in 6-well plates in presence or absence of drug as mentioned above. Cells were then centrifuged at 900 g (2000 rpm) for 5 min. Supernatant was discarded and cells were resuspended in 250 µL lysis buffer and kept at –80°C for at least 4 h before analysis. Total NAD content was measured in cell lysates using a biochemical assay described elsewhere [10]. Cell lysate (20 µL) was plated in a 96-well flat bottom plate. A standard curve was generated using a 1:3 serial dilution in lysis buffer of a β-NAD stock solution. Cycling buffer (160 µL) was added into each well and the plate was incubated for 5 min at 37°C. Ethanol (20 µL), prewarmed at 37°C, was added into each well and the plate was incubated for an additional 5 min at 37°C. Absorbance at 570 nm was read after 5, 10, 15, 20, and 30 min at 37°C on a spectrophotometer. The amount of NAD in each sample was normalized to the protein content for each test sample.

Total ATP cell content was quantified using the ATP determination Kit (Life Technologies, A22066) according to manufacturer’s instructions.

### Plasmids

The lentiviral vector lentiCRISPR [55] was obtained from Addgene (#868, Addgene, ref. no. 52961). The pMD2.G plasmid (#554, Addgene, ref. no. 12259) encodes the envelope of lentivirus. The psPAX2 plasmid (#842, Addgene, ref. no. 12260) encodes the packaging system. LeGOiG2-Bcl-XL (#863) was constructed by subcloning the 771 bp EcoRI fragment from hBcl-XL.dn3 (#274) into LeGOiG2 (#807; Addgene: plasmid 27341).

### Lentivirus production

Recombinant lentiviruses (LentiCRISPRs) were produced as described [56] with the following modification: pMD.G and pCMVDR8.91 were replaced by pMD2.G and psPAX2 respectively.

### Genome editing by CRISPR method

Single guide RNAS targeting the early exon (exon number 2) of PARP1 were chosen in the sgRNA library [57]. LentiCRISPR plasmid specific for PARP1 gene was created according to the provided instructions. Oligonucleotides were designed as follow: Forward 5′-CACCGTTCTAGTCGCCCATGTTTGA-3′; Reverse 3′-AACTCAAACATGGGCGACTAGAAC-5′. Oligonucleotides were synthetized, then phosphorylated and annealed to form oligo complex. LentiCRISPR vector was BsmBI digested and dephosphorylated. Linearized vector was purified and gel extracted and ligated to oligo complex. The lentiCRISPR vector containing the sgRNA was then used for virus production. Cells were infected and selected with the appropriate dose of puromycin (1 µg/ml). Clone isolation was performed by limiting dilution in 96 well-plate.

### TA cloning

TA cloning kit (Life technologies, K202020) was used according to manufacturer’s instructions to sequence DNA fragment containing the region where Cas9 was guided by a sgRNA.

### Immunoblotting

Protein samples were harvested in lysis buffer containing 20 mM HEPES, pH 7.4, 10 mM NaCl, 3 mM MgCl_2_, 2.5 mM EGTA, 0.1 mM dithiothreitol, 50 mM NaF, 1 mM Na_3_VO_4_ or for LC3 expression analysis in a Tris–HCl buffer, pH 7.4, containing 150 mM NaCl, 5 mM EDTA, 1% triton X-100, 2 mM sodium orthovanadate, 0.5 mM phenylmethylsulphonyl fluoride, 0.05% aprotinin (w/v), and 1 mM dithiotreitol. A protease inhibitor cocktail (Roche, 11873580001) was added. Lysates were sonicated and protein concentration was determined using a Bradford assay. Proteins (25–40 μg) were separated by SDS-PAGE on an 8, 10 or 14% polyacrylamide gel, and analyzed by immunoblotting. The mouse anti-PARP1 and the rabbit anti-actin antibodies were from Cell Signaling (9546 and 4970 respectively). After incubation with primary antibody, the following secondary antibodies were applied: polyclonal goat anti-mouse or goat anti-rabbit IgG conjugated with IRDye 680 (LI-COR, B70920-02) or IRDye 800 (LI-COR, 926-32210). Protein bands were visualized using the Odyssey Infrared Imaging System (LI-COR). Odyssey v1.2 software (LI-COR) was used for densitometric analysis. Data were expressed as a percentage of values obtained for control non-treated cells.

### Gene expressions involved in oxidative stress by real-time PCR array analysis PCR analysis

The Human Oxidative Stress Plus RT2 Profiler PCR Array (PAHS-065Z) was used to analyze mRNA levels of 84 key genes related to oxidative stress (antioxidants, ROS metabolism and pathway activity signature genes) in a 96-well format, according to the manufacturer’s instructions (Qiagen). WT or PARP1 KO Jurkat cells were treated with or without APO866 at 10 nM for 40 hours. Reactions were run on a Real-Time PCR system, Bio-Rad CFX96, using RT2 SYBR Green PCR master mix (Qiagen, Switerland). The thermocycler parameters were 95°C for 10 min, followed by 40 cycles of 95°C for 15 s and 60°C for 1 min. Relative changes in gene expression analysis were performed according to QIAGEN Web software using the ΔΔCT method with normalization of the raw data to 5 housekeeping genes. The expression data are presented as real change multiples. Genes with altered expression profile compared to control with fold change value ≥ ±1.5 and *P* < 0.05 were presented.

### Evaluation of cooperative index

To assess a possible synergism between APO866 and Eto (or H_2_O_2_), a cooperative index (CI), was calculated based on the Chou-Talalay method [58–60]. We used the following formula: CI = sum of specific apoptosis of single agent treatment/specific apoptosis of combined treatment. The percentage of specific apoptosis was calculated using the following formula: specific apoptosis = (drug-induced apoptosis – spontaneous apoptosis) / (100 – spontaneous apoptosis) x 100. When CI < 1, CI = 1, and CI > 1, the effects were defined as synergistic, additive, and infra-additive, respectively.

### Statistical analysis

All assays were performed in triplicate and expressed as the mean and standard deviation (SD). All pair-wise comparisons were analyzed by Tukey-Kramer multiple comparisons test or by one-way ANOVA followed by Student’s *t*-test (2-tailed, 2-sample and unequal variance). GraphPad Prism version 6.00 (GraphPad Software, San Diego, CA, USA) was used for statistical analysis. *P* values < .05 were considered statistically significant.

## Abbreviations

7AAD: 7-aminoactinomycin D
ANN: Annexin-V
APF: aminophenyl fluorescein
ATP: adenosine triphosphate
B-CLL: B-chronic lymphocytic leukemia
carboxy-H2DCFDA: 6-carboxy-2′,7′-dichlorodihydrofluorescein diacetate
CI: cooperative index
DAF-2/DA: 5,6-Diaminofluorescein diacetate
ETO: etoposide
HPF: hydroxyphenyl fluorescein
JC-1: 5,5′,6,6′-tetrachloro-1,1′,3,3′-tetraethylbenzimididazolylcarbocyanine iodide
KO: knock-out
L-NAME: N-Nitroarginine methyl ester
MCL: mantle cell lymphoma
NA: nicotinic acid
NAD: nicotinamide adenine dinucleotide
NAD(P)H: dihydronicotinamide-adenine dinucleotide phosphate
NMN: nicotinamide mononucleotide
Nam: nicotinamide
NAMPT: nicotinamide phosphoribosyltransferase
PARP1: poly (ADP-ribose) polymerase-1
RNS: reactive nitrogen species
ROS: reactive oxygen species
